# Identification of genes associated with black rot resistance in cabbage through suppression subtractive hybridization

**DOI:** 10.1007/s13205-015-0311-8

**Published:** 2015-06-23

**Authors:** R. Kaunain Roohie, S. Umesha

**Affiliations:** Department of Studies in Biotechnology, University of Mysore, Manasagangotri, Mysore, 570006 Karnataka India

**Keywords:** Disease resistance, Gene expression, Plant genomics, *Pusa mukta*, Cabbage, Black rot

## Abstract

The suppression subtractive hybridization was employed to elucidate the resistance mechanism in *Brassica oleracea* var. *capitata* upon infection with *Xanthomonas campestris* pv. *campestris*. A cDNA library was constructed enriched in differentially expressed transcripts in the resistant cultivar *Pusa mukta*. A total of 150 unigenes were classified into five functional categories. The present study indicates that the defense-related unigenes accounted for the 35 % of the total unigenes studied. Confirmation of defense-specific representation genes through semiquantitative RT-PCR revealed their increased expression in the resistant cultivar which was validated by qPCR. The resistant cultivar elicited a strong hypersensitive response upon attack by black rot pathogen. The study is first of its kind where the resistant cultivar *Pusa mukta* in India has been assessed for its resistance to the bacterial pathogen.

## Introduction

Black rot, caused by *Xanthomonas campestris* pv. *campestris* (Pammel) Dowson is a major seed-borne biotic constraint for cabbage (*Brassica oleracea* var. *capitata* L.) production by all over the world which causes substantial crop loss especially during warm, humid seasons (Williams 1980). The pathogen enters the plant through the hydathodes and spreads in the vasculature of the leaf and stem. The typical symptom of black rot is the occurrence of V-shaped chlorotic lesions with vertices towards the mid rib of the leaves.

The development and use of black rot-resistant cultivars have long been recognized as important methods of controlling the disease. The inheritance of major gene resistance has been studied in the diploid *B. rapa* (A genome) and in the tetraploids *B. carinata* (BC genome) and *B. napus* (AC genome) (Guo et al. 1991; Ignatov et al. 2000). A single dominant race-specific gene has been mapped to the A genome in *B. napus* (Vicente et al. 2002), and QTLs that control resistance to at least two of the most prevalent races of *X. campestris* pv. *campestris* have been mapped in a Chinese cabbage accession of *B. rapa* (Soengas et al. 2007).

Durable resistance to black rot can be imparted by the genes present in the A and B genomes of *Brassica.* To achieve this aim, genes from the wild relative *Arabidopsis thaliana* could potentially be easier and quicker to characterize molecularly, and either be used directly in transgenic brassica crops, or facilitate the identification and interspecific transfer of homologous black rot resistance genes from A or B genome sources into vegetable crops. Interestingly, most *A. thaliana* accessions are resistant to one or more races of *X. campestris* pv. *campestris*, and more than half exhibit broad-spectrum resistance to all major races of the pathogen suggesting that this wild relative of brassica crops could indeed provide useful sources of durable black rot resistance (Holub 2007). *A. thaliana* and *X. campestris* pv. *campestris* provided one of the earliest experimental models for the investigation of the interactions of *A. thaliana* with a major crop pathogen, the molecular basis of natural variation in black rot resistance is largely unexplored in this pathosystem (Simpson and Johnson 1990).

Defense-related enzymes have a broad action spectrum activity and play key roles in plant–pathogen interactions. Peroxidase is one of the most important factors of the plant’s biochemical defense against pathogenic microorganisms, and is actively involved in the self-regulation of plant metabolism after infection. Peroxidase activity has been associated with pathogenesis, which in turn leads to reinforcement of cell walls with phenolic compounds. Progress in identifying defense mechanisms in cabbage has been very slow, and few defense-related genes in cabbage have been characterized. Functional genomics and proteomics studies in both resistant and susceptible host plants inoculated with *X. campestris* pv. *campestris* will provide key information on the interaction between the bacteria and the host. The control of black rot is still a worldwide threat and development of black rot-resistant cultivars is very essential as only few black rot-resistant cultivars are known worldwide (Vicente and Holoub 2013).

Suppression subtractive hybridization (SSH) is a PCR-based method used to generate unbiased cDNAs library and amplifies only differentially expressed cDNAs. The target cDNA library called as the tester population is denatured, and hybridized to the driver cDNAs population present in the excess. Common fragments to both anneal to each other, whereas tester-specific products remain single stranded represent the differentially expressed genes. SSH is used to generate expressed sequence tags (EST).

The present study involves isolation of genes involved in the resistance to the black rot pathogen in cabbage. The suppression subtractive hybridization method (Diatchenko et al. 1996) was used to generate cDNA libraries enriched in sequences expressed in cabbage leaves during the early stages of hypersensitive response (HR). Several studies indicated that a large number of plant genes are transcriptionally regulated upon challenge by a pathogen, but that most of them may be common to both compatible and incompatible interactions (Maleck et al. 2000). For focusing on genes strictly involved in the hypersensitive response, cDNA from resistant cabbage cv. *Pusa mukta* plants infected with *X. campestris* pv. *campestris* (incompatible interaction) were subtracted with *Pusa mukta* (uninfected). Here we present a catalogue of cabbage non-redundant expressed sequence tags (ESTs) and we describe the identification of genes for which expression is regulated during the early events of *X. campestris* pv. *campestris* infection process. These defense genes provide an important genomic resource for understanding the interactions between cabbage and black rot pathogen. The resistance mechanism of a cabbage cultivar has been assessed for the first time in India at the molecular level.

## Materials and methods

### Screening of cabbage seed samples for the black rot incidence under green house conditions

Seeds of different cabbage cultivars were procured from local traders in Mysore and the cvs. *Pusa mukta* and *Golden acre* from Indian Agricultural Research Institute (IARI), New Delhi, India. All seeds were surface sterilized with 1 % (v/v) sodium hypochlorite solution for 2 min and washed with distilled water thrice. Cabbage cultivars were screened against black rot under green house conditions and the severity of black rot was recorded following 0–5 scale as proposed by Singh et al. (1987). Cabbage seedlings were raised in plastic pots (9 cm diameter) filled with mixture of sterilized soil, sand and farmyard manure (2:1:1). For each cultivar 15 plants in four replicates each were maintained. *Xanthomonascampestris* pv. *campestris* culture was maintained on nutrient starch cycloheximide agar medium (NSCA). The inoculum was prepared and the 15-day-old-seedlings were covered with polythene sheet, 2 h before inoculation to increase humidity. The cabbage seedlings were spray inoculated with *Xanthomonascampestris* pv. *campestris* (1 × 10^8^ cfu/ml).

### Disease scoring

Disease development was monitored by visual examination of the percentage of total symptomatic area. It was assessed for each leaf for the appearance of V-shaped lesions. Measurements were taken upon the first appearance of black rot symptoms (vascular darkening, black rot lesions, and chlorosis), and every 24 h afterward, for a period of 12 days. The following rating scales for the visual disease estimation were employed as per Singh et al. (1987): 0, no visible symptoms (immune I); 1, 1–5 % infection (resistant, R); 2, 6–15 % infection (moderately resistant MR); 3, 16–30 % infection (moderately susceptible, MS); 4, 31–50 % infection (susceptible, S) and 5, >50 % infection (highly susceptible, HS).

### Suppression subtractive hybridization

Cabbage leaves were harvested at 12, 24 and 48 h post-inoculation (hpi) with *Xanthomonascampestris* pv. *campestris* (UOMBT-6 isolate), either eliciting an incompatible interaction or a compatible interaction and immediately stored in liquid nitrogen. Total RNA was isolated using the RNeasy Plant kit (Qiagen, France). RNA yield was determined by measuring the absorbance at 260 nm in a UV–visible spectrophotometer (Hitachi, U-2000, Tokyo, Japan) and RNA integrity was checked by electrophoresing 3 µg of total RNA through 1.2 % agarose gel. Poly (A)^+^ RNA was purified from total RNA using mRNA purification kit (BangaloreGenei, Bangalore, India).

Suppression subtractive hybridization was performed as described by Diatchenko et al. (1996) using PCR select cDNA subtraction kit (BD biosciences Clonetech, Palo Alto, CA, USA). Approximately 400 ng of tester and driver cDNA was digested with Rsa I in a 40 µl reaction mixture containing 30 units of enzyme for 4 h at 37 °C. The restricted cDNA fragments were purified using a MinElute reaction clean up kit (Qiagen, France) and eluted from MinElute columns in 10 µl sterile water. Digested tester cDNA (1 µl) was diluted in 5 µl of water. A 2 µl aliquot of the diluted tester cDNA was then either ligated to 2 µl of adaptor 1 (10 µM) or 2 µl of adaptor 1 (10 µM) in separate ligation reactions in a total volume of 10 µl at 14 °C overnight using two units of T_4_ DNA ligase (BD Biosciences clonetech) in the buffer supplied by the manufacturer. After ligation reactions were heated at 72 °C for 5 min to inactivate the ligase.

Thereafter 1.5 µl of ds cDNA together with 1 µl hybridization buffer (BD Biosciences clonetech) was added to each of two tubes containing 1.5 µl of adapter 1 and adapter 2R ligated tester cDNA (1:10) diluted, respectively. The solution was overlaid with mineral oil, the DNA was denatured (1.5 min, 98 °C) and then allowed to anneal for 12 h at 68 °C. After this first hybridization, the two samples were combined and a fresh portion of heat-denatured driver (100 ng) in 1 µl hybridization buffer was added. The sample was left to hybridize for an additional 16 h at 68 °C for 7 min and stored at −20 °C until use. The final ratio of the tester to driver in both the forward and reverse subtraction experiments was 300:1. Six separate suppressive PCR amplification reactions were performed for the forward and reverse subtracted cDNA samples.

### cDNA library construction and amplification of cDNA inserts

Suppression subtractive hybridization was performed as described by Diatchenko et al. (1996) using PCR select cDNA subtraction kit (BD biosciences Clonetech, Palo Alto, CA) according to the manufacturer’s instructions. The cDNA obtained from *Xanthomonascampestris* pv. *campestris* inoculated *Pusa mukta* cultivar at 12, 24 and 48 hpi (hours post-inoculation) was used as “tester” population and cDNA from the uninoculated *Pusa mukta* was used as “driver” population. The efficiency of subtraction was analyzed by comparing cDNA abundance before and after subtraction by PCR using specific primers for the constitutively expressed cabbage-specific 18S rRNA gene (Table 1). PCR amplification was performed using *Taq* DNA polymerase (BangaloreGenei, Bangalore, India) and 5 µl aliquots were removed following determined numbers of PCR cycles. The amplified products were examined in 2 % agarose gel. The differences in the number of cycles, which were needed to generate an approximately equal amount of the corresponding PCR product in subtracted and unsubtracted samples, served to indicate the subtraction efficiency.

**Table 1:** List of primers used for the gene expression studies

Gene	Forward primer	Reverse primer
18S rRNA	GCTACGCAGAAGACAGTTGAT	TGGGCACACGGAAGGACATAC
Peroxidase	ATGGCTGAGGAGTCTCCTC	TCCAGTAGAGTATCCTTCTCG
Catalase	GGAAGCCTACTTGTGGAATCA	GAACCTTCTCAGCACATCTAA
Superoxide dismutase	GGTACGGAAGAGTACACAGAAC	CGACGCAAATCCAAACACATAA

### Cloning of the subtracted cDNA

The secondary PCR products were purified using the QIAquick PCR purification kit (Qiagen, France). The subtracted cDNA fragments were cloned into the pGEMT-easy using a pGEMT-easy cloning kit (Promega, USA) and transformed into *Escherichia coli* JM109 cells. Individual colonies containing recombinant plasmids were inoculated into 100 µl Luria–Bertani broth in 96 well microtitre plates. Cultures were grown overnight at 37 °C with gentle shaking (100 rpm). 100 µl of 15 % glycerol was added to each of the wells and the microtitre plates were stored at −80 °C until use.

### Identification of insert size of cDNA by PCR amplification

Sequencing of cDNA clones was performed using T7 or SP6 primer. In order to correct the sequencing amibiguities, the sequences were edited by removing the plasmid and SSH adaptor sequences. The edited sequences were used to query the NCBI (National Center for Biotechnology Information, USA) databases using the blastX, blastN and dbEST algorithms (Altschul et al. 1990). The cDNA were classified according to the *E*-values generated in the searches. Sequences were checked for stop codons to ensure that cDNA fragments represented a position of open reading frame.

### Enzyme studies

#### Preparation of crude enzyme extracts

Seeds of all the cabbage cultivars were plated onto moist blotter discs placed in a 9-cm-diameter petri dish, at a density of 25 seeds per plate following standard procedures of the International Seed Testing Association (ISTA 2003). The plates were incubated at 28 ± 2 °C for 8 days until the cotyledons completely opened. The 12-day-old seedlings were spray inoculated with *X.campestris* pv. *campestris* (UOMBT-6). The seedlings were harvested 48 h post-inoculation (hpi) and frozen in liquid nitrogen and stored at −80 °C until further use.

Cabbage seedlings (1 g) were macerated to a fine paste in a pre-chilled mortar with 50 mM phosphate buffer (pH 8.8) (w/v; 1:1). The homogenate was centrifuged at 10,000 rpm for 10 min at 4 °C and the supernatant was used directly for enzyme assay. The homogenate was centrifuged at 12,000 rpm for 20 min at 4 °C and the supernatant served as enzyme source for peroxidase (POX) and superoxide dismutase (SOD).

#### Peroxidase assay

Peroxidase (POX) activities were measured at room temperature according to the standardized procedure of He et al. (2001). The POX assay reaction mixture contained 7.5 μL of 10 mM guaiacol in 50 mM sodium phosphate buffer (pH 6.0), 100 μl of crude extract, 792.5 μl of 10 mM sodium phosphate buffer (pH 6.0), and 100 μl of 600 mM H_2_O_2_. The change in optical density at 470 nm was measured for 1 min. POX activity was calculated as change in absorbance units min^−1^ mg^−1^.

#### Superoxide dismutase assay

Total superoxide dismutase (SOD) activity was assayed based on the method of Beauchamp and Fridovich (1971) as described by Madamanchi and Alscher (1991) by measuring the ability of the enzyme to inhibit the photochemical reduction of nitro-blue tetrazolium. The 3 ml reaction mixture contained 50 mM potassium phosphate buffer (pH 7.8), containing 0.1 mM EDTA, 13 mM methionine, 75 mM nitro-blue tetrazolium, 2 mM riboflavin, and the tissue extract. Riboflavin was added last, the tubes were stirred and the reaction was initiated by placing the tubes under two 15 W fluorescent lamps. The reaction was terminated after 10 min by switching off the light. Non-illuminated tubes served as a blank. Blue color was measured at 560 nm. Activity was expressed in enzyme units per mg protein. The volume of enzyme extract corresponding to the 50 % inhibition of the reaction was considered as one enzyme unit.

#### Catalase activity assay

Catalase (CAT) activity was determined by measuring the rate of decrease in absorbance at 240 nm of a solution of 30 Mm H_2_O_2_ in 50 mM potassium phosphate buffer (pH 7.0) at 25 °C (Bunaurio 1987). Activity was expressed in enzyme units per mg protein. One unit is defined as the amount of enzyme catalyzing the decomposition of 1 mmol H_2_O_2_ min^−1^ calculated from the extinction coefficient for H_2_O_2_ at 240 nm of 0.036 cm^2^ mmol.

### Temporal pattern of enzymes

Seeds of all the cabbage cultivars were plated onto moist blotter discs placed in a 9-cm-diameter petri dish, at a density of 25 seeds per plate following standard procedures of the International Seed Testing Association (ISTA 2003). The plates were incubated at 28 ± 2 °C for 8 days until the cotyledons completely opened. The temporal pattern of POX, SOD and CAT was studied in two different cultivars, that were resistant (*Pusa mukta*) and highly susceptible (cv. *NBH boss*) based on disease incidence under greenhouse conditions. The cabbage seedlings raised as above were harvested at different time intervals: 0, 3, 6, 9, 12, 15, 18, 21, 24 up to 60 h after pathogen inoculation (hpi) and subjected to enzyme estimations as explained previously. Samples inoculated with distilled water served as control.

### Protein estimation

Protein content of the extracts for all the estimated enzymes was determined using the standard procedure of Bradford (1976) with BSA (Sigma, USA) as standard.

### Native-PAGE analyses of POX, SOD and CAT

The isoforms profiles of POX, SOD and CAT were examined by discontinuous native polyacrylamide gel electrophoresis (native-PAGE) following the procedure of Laemmli (1970) and Yang et al. (1999) with slight modifications, respectively. Enzyme extracts (60 mg protein) of R and HS cabbage cultivars at 12, 18 and 15 h of both inoculated and control were loaded onto 8 % (w/v) polyacrylamide gels with a vertical mini-gel electrophoresis unit (Biometra, Gottingen, Germany). The electrode buffer was Tris base (6.0 g Tris base, 14.4 g glycine and 1 l distilled water). Electrophoresis was performed at a constant voltage of 50 V initially for 1 h and 100 V to complete electrophoresis.

#### Activity staining for POX

After electrophoresis, POX isoforms were visualized by soaking the gels in staining solution containing 100 mg benzidine dissolved in 1 ml of absolute alcohol and made up to 40 ml using distilled water. Clear solution was obtained by adding 500 ml of glacial acetic acid to the above mixture and undissolved particles of benzidine were removed by filtering the solution through cotton. H_2_O_2_ (250 ml) was added to the filtered solution at the end and gels were incubated in the solution until bands appeared (Schrauwen 1966).

#### Activity staining for SOD

After electrophoresis, SOD isoforms were visualized by completely submerging the gel in freshly prepared staining buffer containing 50 mM phosphate buffer, 0.1 ml EDTA, 28 mM TEMED, 0.003 mM riboflavin and 0.25 mM nitroblue tetrazolium for 30 min in dark condition. Thereafter, the gel was placed on an illuminated glass plate until the bands become visible.

#### Activity staining for CAT

CAT activity was performed following the procedure of Yang et al. (1999). The gel was first rinsed three times with distilled water and then incubated in 0.003 % H_2_O_2_ for 10 min. The gel was then stained with 2 % ferric chloride and 2 % potassium ferricyanide; when chromatic bands appeared, the stain was drained off the gel and the gel was rinsed thoroughly to stop the reaction and the washed with distilled water. Achromatic bands demonstrated the presence of CAT activity.

### Statistical analysis

All the experiments were performed twice with similar results. The data obtained from green house experiments were analyzed separately for each experiment and were subjected to two-way analysis of variance (ANOVA) using the statistical software SAS (version 9.0). The means were compared for significance using Fisher’s LSD. Significant effects of pathogen inoculation on enzyme activities were determined by the magnitude of *F* value (*p* = 0.05).

### Semiquantitative RT-PCR for POX, SOD and CAT

Total RNA of resistant (cv. *Pusa mukta*) and susceptible (cv. *NBH boss*) cultivars was isolated from leaf samples of 12, 24 and 48 hpi with *X.campestris* pv. *campestris* using plant RNeasy kit (Qiagen, France). The mRNA was purified using mRNA purification kit (Bangalore Genei, Bangalore, India). One-step reverse transcription-PCR (RT-PCR) was performed using *M*-*MuLV* reverse transcriptase (BangaloreGenei, Bangalore, India) according to the manufacturer’s instructions. First-strand cDNA was diluted (1:5) with RNase-free water and used as a template for PCR. The RT-PCR primers were designed from the selected unigenes using primer Quest Integrated DNA Technologies (Table 1).

Cabbage 18S rRNA was used as endogenous control. Semiquantitative RT-PCR was carried out and the PCR cycling parameters consisted of 94 °C for 2 min, annealing at 58 °C for 1 min and extension at 72 °C for 2 min and a final extension at 72 °C for 5 min followed by 16 cycles of POX, 20 cycles for SOD, 22 cycles for CAT and 25 cycles for 18S rRNA.

### Quantitative real-time PCR

Primer pairs specific to POX, SOD and CAT were designed for qRT-PCR from the sequence obtained from the SSH library. The primers were checked for specificity by semiquantitative RT-PCR. Each reaction consisted of 10 µl consisting of 5 µl of SYBR green PCR master, 2 µl nuclease-free water and 1 µl of reverse transcribed cDNA product, qRT-PCR was performed using single one real-time PCR system (Roche, Switzerland). The thermocycler program had an initial 95 °C for 10 min followed by 40 cycles consisting of 30 s denaturation at 95 °C, 60 s annealing and extension at 60 °C. At the end of each reaction, a melting curve analysis (64–95 °C with a heating rate of 0.5 °C/s) was performed to determine the specificity of the reaction.

## Results

### Screening of cabbage seed samples for the black rot incidence under greenhouse conditions

The ten cabbage cultivars used in the study were classified based on the appearance of black rot incidence which ranged between 4 and 66 %. The *Pusa mukta* cultivar showed 3 % disease incidence and hence considered as the resistant cultivar, five cultivars (*Indam krishna, Gaurav, Indam saina, Unnati and NS 43*) were moderately resistant (6–14 %), two cultivars (*Golden acre, Quisto*) were susceptible (30–33 %) and two cultivars (*F1 bhima and NBH boss*) were highly susceptible (63–66 %) to black rot disease under green house conditions (Table 2).

**Table 2:** Screening of cabbage cultivars for the presence of black rot disease under green house conditions

Cultivars	Incidence of black rot (%)	Categorization
Pusa mukta	4 ± 0.2	R
Indam krishna	14 ± 0.2	MR
Gaurav	9 ± 0.3	MR
Indam saina	13 ± 0.4	MR
Unnati	9 ± 0.3	MR
NS 43	9 ± 0.4	MR
Golden acre	44 ± 0.8	S
Quisto	43 ± 0.6	S
F1 bhima	63 ± 0.7	HS
NBH boss	66 ± 0.8	HS

Values are the mean ± SE of four replicates and repeated thrice

### Construction of subtracted library

Cabbage leaf samples of resistant cultivar *Pusa mukta* were harvested at 12, 24 and 48 hpi and used to identify differentially expressed genes upon inoculation with *X.campestris* pv. *campestris*. A cDNA subtractive library was constructed wherein *X. campestris* pv. *campestris* inoculated leaf samples acted as the tester and uninoculated leaf samples as driver. The subtraction efficiency was evaluated by expression of the 18S rRNA between subtracted and unsubtracted cDNAs. The amount of 18S rRNA decreased significantly after subtraction and could be detected in agarose gel at 28 PCR cycles whereas in the unsubtracted samples 18SrRNA was detected at 18 PCR cycles. This indicated that the subtraction had worked well. Finally 1000 clones were obtained and 500 clones were randomly selected from the library and the insert size was detected by PCR with SSH primer provided in the subtraction kit. The 300 positive clones carrying single exogenous fragment were detected and the length of the inserted fragments ranged from 200 to 700 bp.

### Analysis of EST sequences

**Fig. 1:**
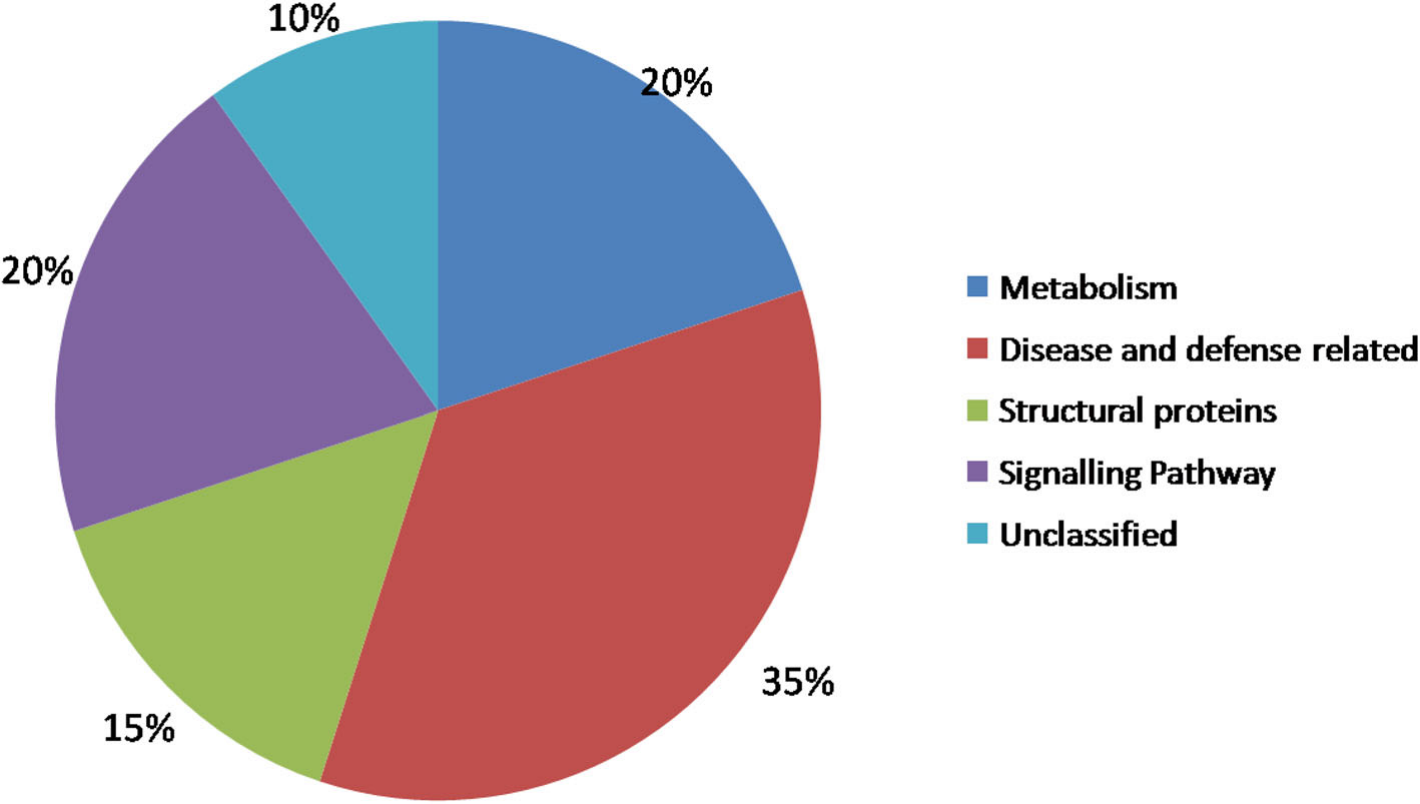
EST classification of putative biological function based on homologies to sequences of known function detected by BLASTN searches

The 150 unigenes obtained from the 300 positive clones were classified into five major categories: metabolism, disease and defense-related, structural proteins, signaling pathway related and unclassified group. The metabolism-related unigenes accounted for 20 %, defense related (35 %), structural proteins (15 %), signaling pathway related (20 %) and 10 % were with unknown function or did not show any significant similarity with known genes or hypothetical proteins (Fig. 1).

All the unigenes were subjected to similarity search using BLASTX, BLASTN dbEST databases. Sequences showing the *E*-value <10^−3^ were considered as significant. The most abundant group consisted of the defense-related genes catalase, peroxidase, HSP70, superoxide dismutase were prominent which are predicted to play an important role in the resistance process upon pathogen attack followed by the metabolism and structural proteins category. About 10 % of the unigenes could not be functionally categorized (Table 3). The black rot-inducible genes in cabbage obtained from the SSH cDNA library submitted dbEST with library name: LIBEST_028420 *Brassica olerace*a var. *capitata* SSH Library with the accession nos JZ585251 to JZ585260 and JZ585519 to JZ585523, respectively.

**Table 3:** List of black rot-inducible genes in cabbage obtained from the SSH cDNA library matched upon BLAST analyses with plant genes of known functions

Clone no.	BLAST/similarity	Related accession no
Metabolism		
Pm57	Ribulose phosphate	GQ184377.1
Pm014	Photosystem I	P06512
Pm074	Photosystem II	AY185358.2
Pm10	Sucrose synthase	AA41682
Pm092	4-Hydroxy phenyl pyruvate	AF251665
Pm043	ADP-glucose pyrophosphorylase	AF010283
Pm022	Starch debranching enzyme precursor	BAA09167
Disease/defense related		
Pm099	WRKY transcription factor	GQ168839.1
Pm064	Super oxide dismutase	AF071112.1
Pm062	Cytochrome P450	AY029178.1
Pm019	Heat shock proteins	AAB97316.1
Pm204	Glutathione peroxidase	AF411209.1
Pm301	Beta glucosidase	NP199277
Pm299	MAP- kinase like protein	AAX96170
Pm350	Peroxidase	GR723799.1
Pm401	Catalase	AB474628.1
Pm435	Superoxide dismutase	EU186343.1
Pm415	Lipoxygenase	GR724065.1
Structural proteins		
Pm025	40S ribosomal proteins	AF144752.1
Pm009	60S Ribosomal proteins	L21897.1
Pm 046	DNA polymerase sub unit	Up
Pm089	Membrane proteins	U13631.1
Pm333	Translation initiation factor	Z21510.1
Signaling proteins		
Pm033	Pyruvate dehydrogenase	JF682847.1
Pm007	Phosphatase	FJ346565.1
Pm050	Zinc finger protein	HM579881.1
Pm024	ABC transporter like protein	DQ296184.1
Pm045	Serine/threonine kinase gene	DQ375116
Unclassified		
Pm082	Hypothetical protein 1	
Pm092	Non specific disease resistance 1	
Pm002	Hypothetical protein 2	

### Enzyme studies

#### Temporal pattern

**Fig. 2:**
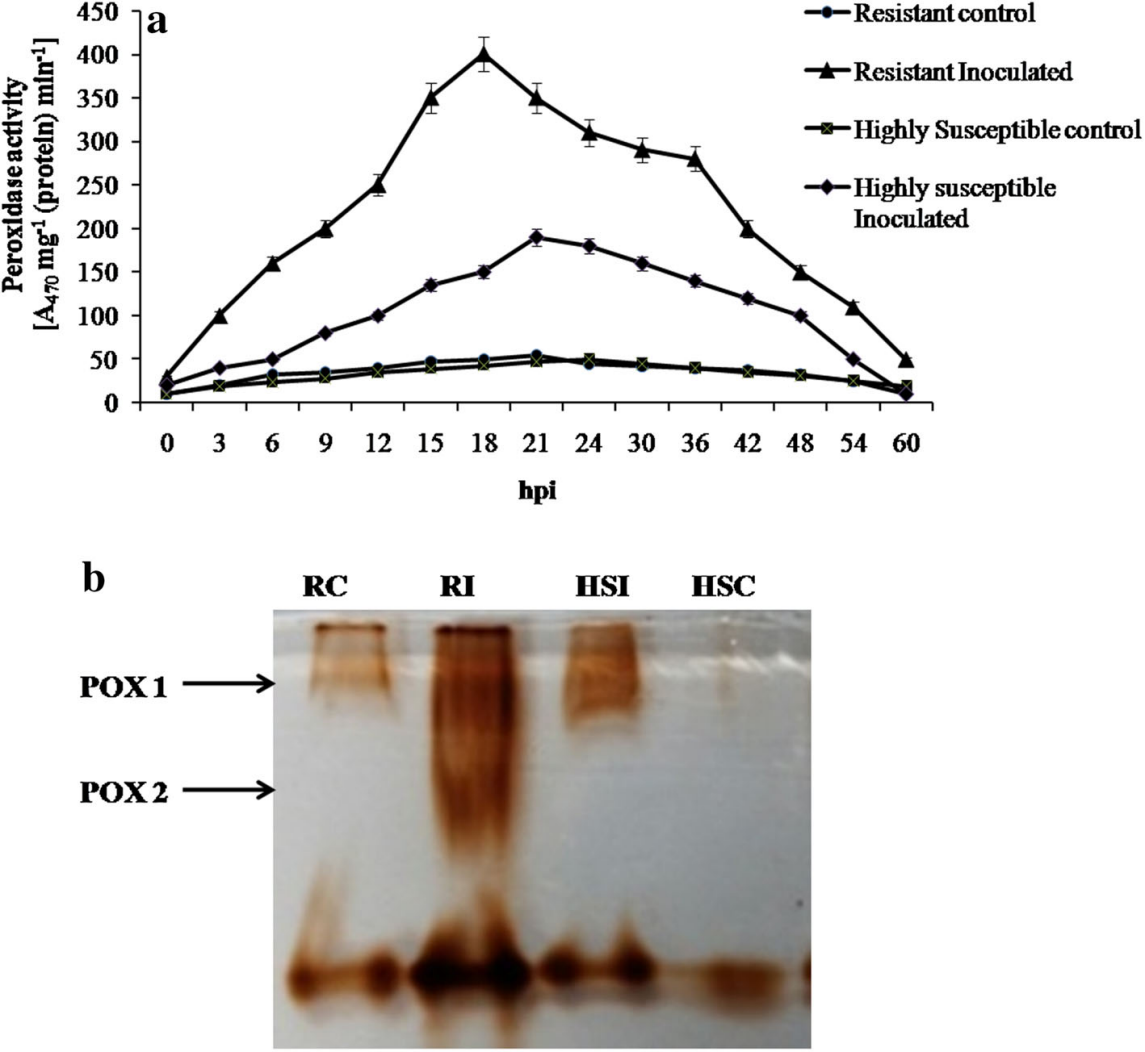
**a** Temporal pattern of POX activity in resistant (*Pusa mukta*) and highly susceptible (*NBH boss*) cultivars with and without pathogen inoculation. The data expressed as the average of three independent experiments with three replicates each. *Bars* indicate standard error. **b** Differential expression of isoforms of POX in resistant (*Pusa mukta*) and highly susceptible (*NBH* *boss*) cabbage cultivars with and without pathogen *X. campestris* pv. *campestris*. Each lane was loaded with 100 µg protein. *RC* resistant control, *RI* resistant inoculated, *HSC* highly susceptible control, *HSI* highly susceptible inoculated

Temporal study was undertaken to estimate the POX, SOD and CAT enzyme activities at regular intervals from 0 to 60 h and the peak activities were noted at 12, 18 and 15 h for POX, SOD and CAT, respectively. The POX activity was high at 12 h after pathogen inoculation (hpi) in resistant cultivar *Pusa mukta*. The activity found to be 1.8 units at 12 h, whereas in highly susceptible cultivar the POX activity was 0.1 units at 15 hpi. This was proved by native-page analysis wherein two isoforms of POX were expressed in resistant cultivar as compared to HS (Fig. 2a, b).

**Fig. 3:**
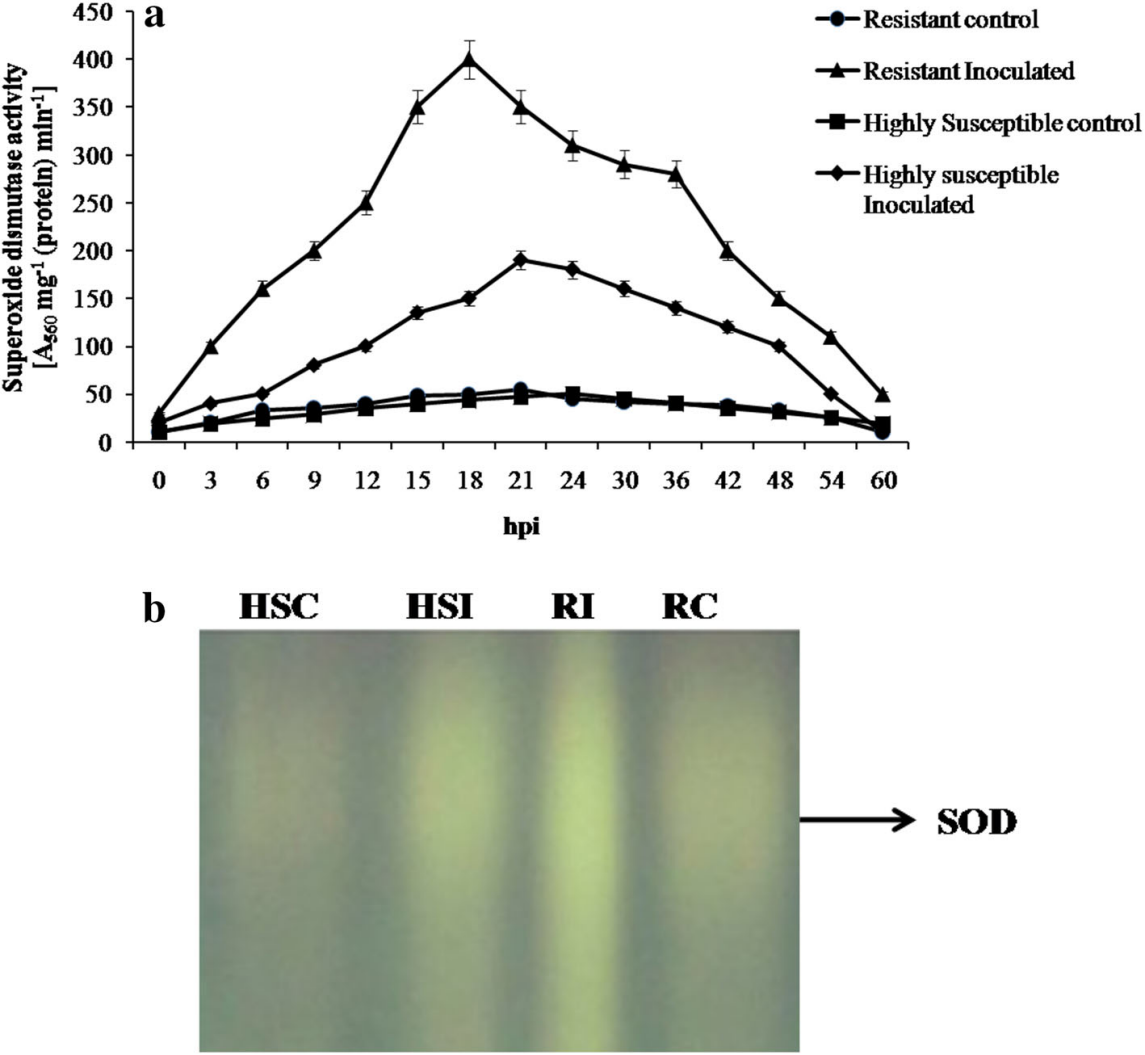
**a** Temporal pattern of SOD activity in resistant (*Pusa mukta*) and highly susceptible (*NBH boss*) cultivars with and without pathogen inoculation. The data are expressed as the average of three independent experiments with three replicates each. *Bars* indicate standard error. **b** Differential expression of isoforms of SOD in resistant (*Pusa mukta*) and highly susceptible (*NBH boss*) cabbage cultivars with and without pathogen *X. campestris* pv. *campestris*. Each lane was loaded with 100 µg protein. *RC* resistant control, *RI* resistant inoculated, *HSC* highly susceptible control, *HSI* highly susceptible inoculated

The SOD activity was high at 18 h after pathogen inoculation (hpi) in resistant cultivar *Pusa mukta*. The activity found to be 400 units at 18 h, whereas in highly susceptible cultivar the SOD activity was 190 units at 21 hpi. This was proved by native-page analysis wherein SOD activity was high in resistant and a smear was observed in HS cultivar (Fig. 3a, b).

**Fig. 4:**
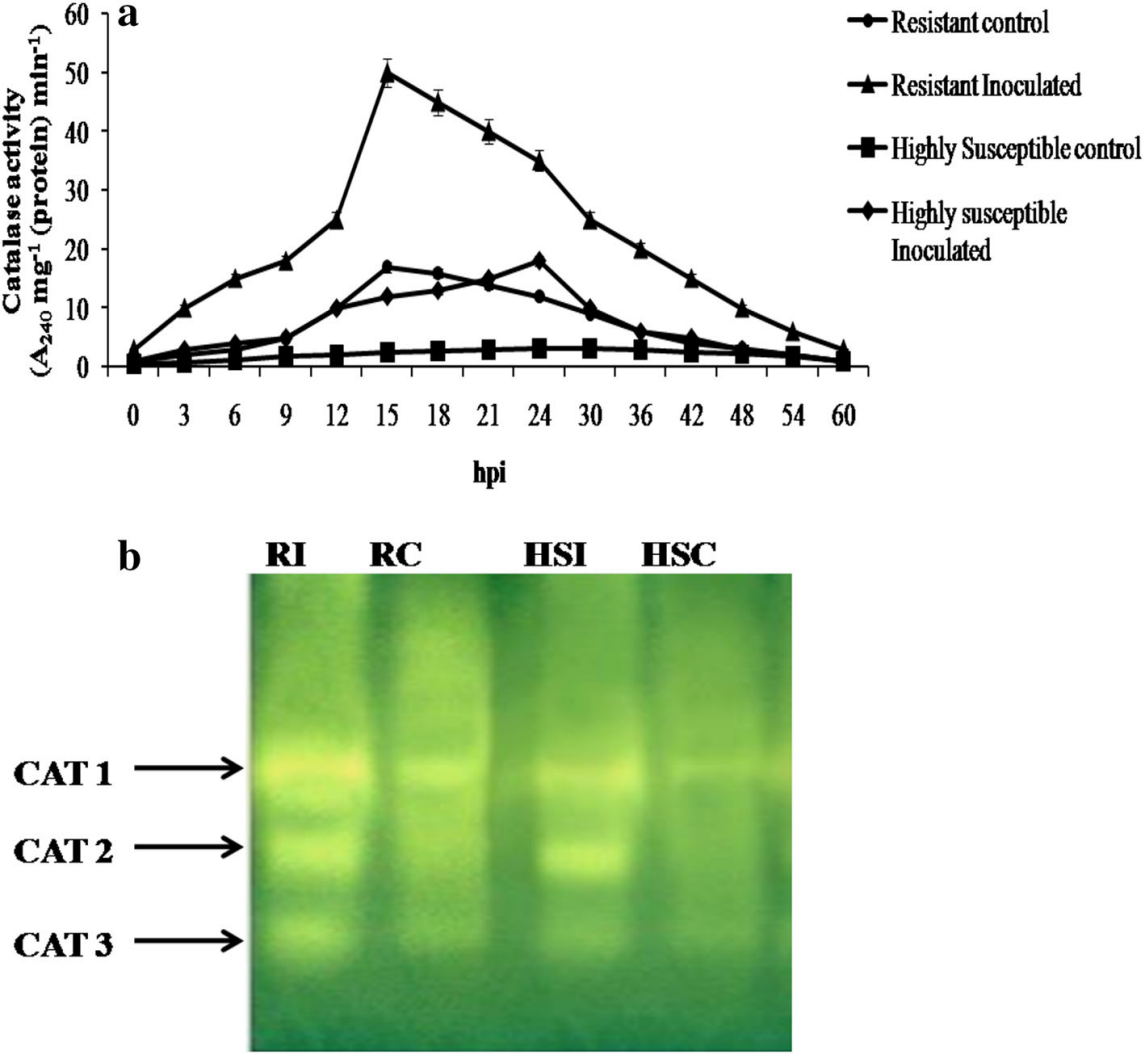
**a** Temporal pattern of CAT activity in resistant (*Pusa mukta*) and highly susceptible (*NBH boss*) cultivars with and without pathogen inoculation. The data are expressed as the average of three independent experiments with three replicates each. *Bars* indicate standard error. **b** Differential expression of isoforms of CAT in resistant (*Pusa mukta*) and highly susceptible (*NBH boss*) cabbage cultivars with and without pathogen *X. campestris* pv. *campestris*. Each lane was loaded with 100 µg protein. *RC* resistant control, *RI* resistant inoculated, *HSC* highly susceptible control, *HSI* highly susceptible inoculated

The temporal changes in CAT showed peak activity at 15 hpi in resistant cultivar with 50 units as compared to HS which had an activity of 15 units at 24 hpi. The native page revealed that the R showed three distinct isoforms upon pathogen attack, whereas the uninoculated control showed only two isoforms. But only one isoform was observed in HS untreated (Fig. 4a, b).

### Semiquantitative RT-PCR

**Fig. 5:**
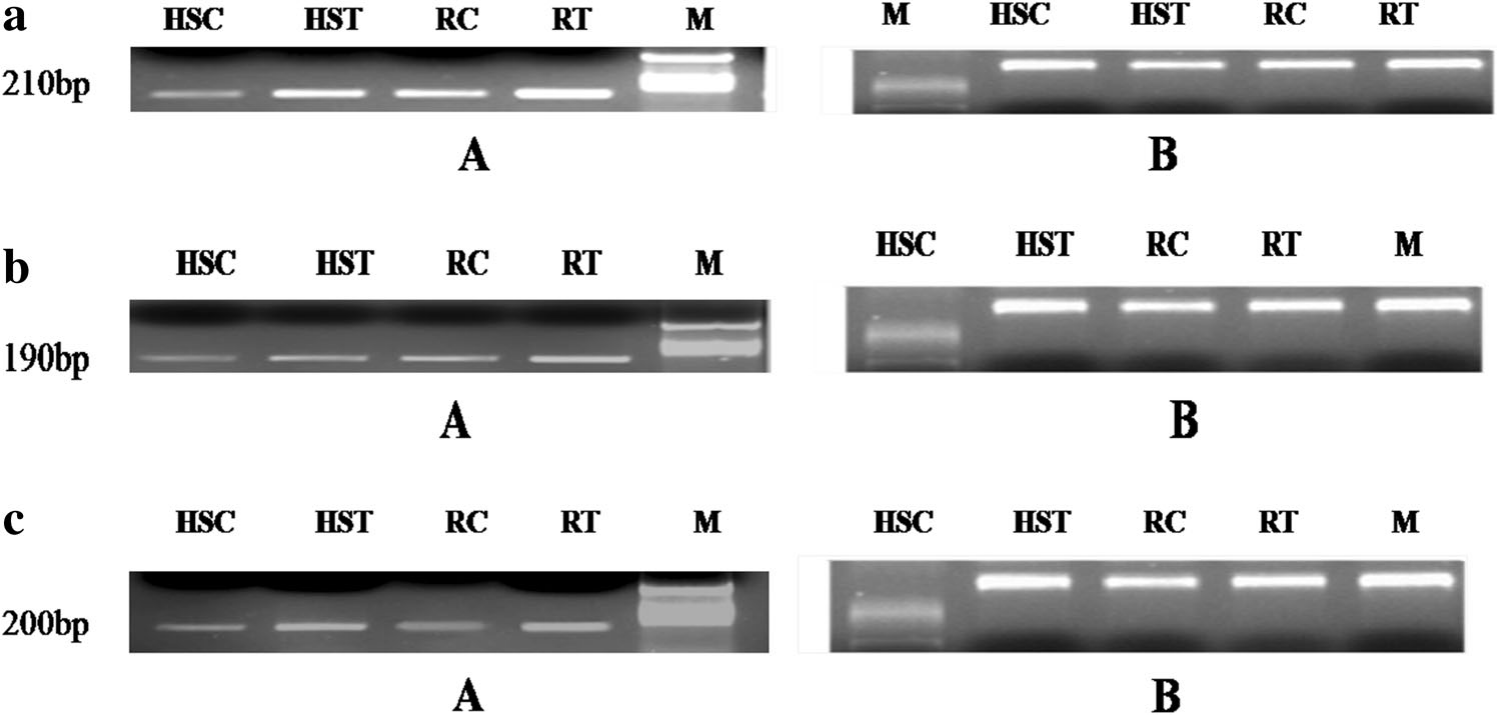
**a** Semiquantitative PCR to study POX gene expression upon pathogen inoculation in highly susceptible (HS) and resistant (R). *A* PCR products assayed by electrophoresis (1.5 % agarose gels) stained with ethidium bromide. *HSC* HS control, *HST* HS + pathogen, *RC* R control, *RT* R + pathogen, *M* ladder (100 bp). *B* 18S rRNA internal control, *R* resistant (cv. *Pusa mukta*), *HS* highly susceptible (cv. *NBH boss*). **b** Semiquantitative PCR to study SOD gene expression upon pathogen inoculation in highly susceptible (HS) and resistant (R). *A* PCR products assayed by electrophoresis (1.5 % agarose gels) stained with ethidium bromide. *HSC* HS control, *HST* HS + pathogen, *RC* R control, *RT* R + pathogen, *M* ladder (100 bp). B-18S rRNA internal control, *R* Resistant (cv. *Pusa mukta*), *HS* Highly susceptible (cv. *NBH boss*). **c** Semiquantitative PCR to study CAT gene expression upon pathogen inoculation in highly susceptible (HS) and resistant (R). *A* PCR products assayed by electrophoresis (1.5 % agarose gels) stained with ethidium bromide. *HSC* HS control, *HST* HS + pathogen, *RC* R control, *RT* R + pathogen, *M* ladder (100 bp). *B* 18S rRNA internal control, *R* resistant (cv. *Pusa mukta*), *HS* highly susceptible (cv. *NBH boss*)

Three defense-related unigenes, peroxidase, superoxide dismutase and catalase showed high homology percentage and were selected to study their expression in resistant and susceptible cultivars during infection with *Xanthomonascampestris* pv. *campestris.* The transcript level was investigated at 24 and 48 hpi. The genes were compared with the internal control being 18S rRNA. The 18S rRNA was expressed in both cultivars. The expression of the defense genes was higher in resistant cultivar. The expression of peroxidase was higher in the resistant cultivar at 48 hpi compared to the susceptible cultivar. The expression of catalase was detected in both the susceptible and resistant cultivar, but the expression was relatively higher in the resistant cultivar (Fig. 5a–c).

### Quantitative real-time PCR

**Fig. 6:**
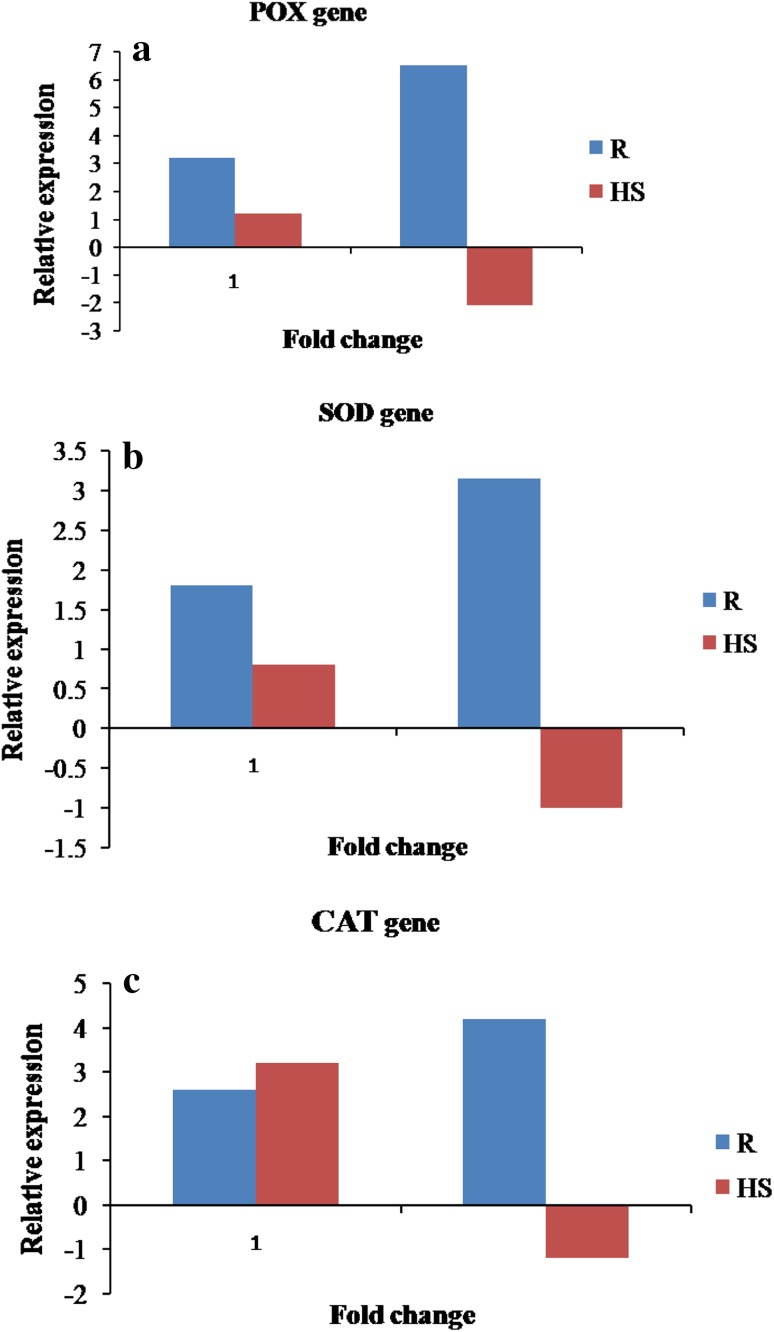
**a** Quantitative real-time PCR assay for relative expression levels of POX in both resistant and highly susceptible cultivars upon inoculation with *X. campestris* pv. *campestris*. Values are the means of *n* = 3. *R* resistant (cv. *Pusa mukta*), *HS* highly susceptible (cv. *NBH boss*). **b** Quantitative real-time PCR assay for relative expression levels of SOD in both resistant and highly susceptible cultivars upon inoculation with *X. campestris* pv. *campestris*. Values are the means of *n* = 3. Resistant (cv. *Pusa mukta*); *HS* highly susceptible (cv. *NBH boss*). **c** Quantitative real-time PCR assay for relative expression levels of CAT in both resistant and highly susceptible cultivars upon inoculation with *X. campestris* pv. *campestris*. Values are the means of *n* = 3. *R* resistant (cv. *Pusa mukta*), *HS* highly susceptible (cv. *NBH boss*)

Quantitative real-time PCR revealed that the resistant cabbage cultivar POX gene recorded the relative gene expression of 3.2 in control which up regulated to 6.5 upon pathogen inoculation. In the case of highly susceptible it is down regulated to −2.1 fold upon infection by *X. campestris* pv. *campestris*. The HS control showed a POX expression of 1.2-fold (Fig. 6a). The relative gene expression of SOD gene in resistant cv. 1.8 in control which up regulated to 3.13 upon pathogen inoculation. In the case of highly susceptible down regulated to −1 fold upon infection by *X. campestris* pv. *campestris*. The HS control showed a POX expression of 1.0-fold (Fig. 6b).

The relative gene expression of CAT gene in resistant cv. 2.6 in control which up regulated to 4.2 upon pathogen inoculation. In the case of highly susceptible down regulated to −1.2 fold upon infection by *X. campestris* pv. *campestris*. The HS control showed a POX expression of 3.0 fold (Fig. 6c).

## Discussion

SSH which is a powerful technique used to enrich libraries with differentially expressed cDNAs and includes a normalization step that enables detection of low abundance differentially expressed transcripts such as those involved in signaling and signal transduction and might thus identify essential components in biological processes (Birch and Kamoun 2000). The transcriptional diversity in *B. oleracea* var. *capitata*–*X*. *campestris* pv. *campestris* interaction in resistant cultivar *Pusa mukta* was studied. 150 unigenes were obtained from the 500 randomly picked positive clones. Upon functional annotation of the 150 unigenes, the ESTs with known biological function accounted for 80 % of total genes. The main aim of the study was to focus on the genes involved in resistance mechanism against *X. campestris* pv. *campestris* in resistant cultivar *Pusa mukta*. *Pusa mukta* is a resistant cultivar obtained from a cross between EC 24855 × EC 10109 by pedigree selection. It is one of the popular resistant cultivar in northern part of India, but its performance depends on the geographic location and its performance in the southern part of India was assessed.

Some reports of up-regulation of defense genes during several plant–pathogen interactions exist in the literature (Fernandez et al. 2004), suggesting the role of proteins in plant defense mechanisms. It is also established fact that reactive oxygen species (ROS) are the main source of damage to cell under stress conditions. ROS scavengers like superoxide dismutase, catalase and peroxidase are produced in significant quantity to combat the ROS produced during stress. Peroxidase accumulation and lignifications occur predominantly in the hydathodes, and to a greater extent in resistant cabbage varieties than in susceptible ones. Total peroxidase activities in hydathodal fluids were noted between resistant and susceptible cabbage varieties during pathogenesis.

The accumulation of peroxidases in the hydathodal fluids of cabbage varieties correlates positively with the level of varietal resistance to black rot. Accumulations of peroxidase in hydathodal fluids are also positively correlated with the occurrence of lignification in the hydathodal region. These results suggest that peroxidases have a dual role in black rot resistance: direct antagonism of *X. campestris* pv. *campestris* at the site of infection, as well as indirect suppression of pathogen spread via peroxidase-mediated increases in lignifications (Gay 2000). The roles of hydrogen peroxide (H_2_O_2_)-protective genes (katA, katG, and ahpC) and a peroxide sensor/transcription regulator (oxyR) in the viability of *X. campestris* pv*. campestris* at an elevated temperature were evaluated when exposed to multiple stresses in the environment and during interaction with a host plant with *Xanthomonas campestris* pv. *campestris* (Sarinya et al. 2011). The study highlights the role of catalase in *Xanthomonas campestris* pv. *campestris.*

The NDR1 (non-race-specific disease resistance) protein is a key component of the signaling pathway of many NBS-LRR resistance proteins. In the *Pseudomonas syringae*–*A. thaliana* interaction, NDR 1 expression is induced in response to pathogen challenge. WRKY—a transcription factor was putatively encoded. The promoter element (W-box) that binds WRKY proteins has been found in several *A. thaliana* genes, which exhibited common regulation patterns under different systemic acquired resistance (SAR)-inducing or -repressing conditions (Maleck et al. 2000; Fernandez et al. 2004).

HSP70 is one of the major classes of chaperone molecules and also functions in the response to pathogen attack. Therefore, HSP70 is an essential component of the hypersensitive response defense mechanism in plants. Mitogen-activated protein kinases (MAPK) are important intracellular mediators of the information in early defense signaling through protein phosphorylation of downstream signaling components and targets several transcription factors. Moreover, our results indicated that there was a negative relationship between catalase and peroxidase activity with SOD activity. We have identified 150 unigenes from cabbage by cDNA-SSH, representing 10 % of which are novel genes. The presence of cDNAs with non-significant matches are due to the high cut-off value which was set to increase the level of significance of matches with sequences in the databases or that these proteins/genes have not been functionally characterized yet in any plant species. Excessive ROS causes oxidative damage to the plant cell structures, nucleic acids, lipids and proteins under various biotic stress conditions. Hence the up-regulation of ROS-scavenging enzymes related transcripts such as SOD, POD and CAT transcripts in our library suggests that it protects the cell membrane. These results are in conjuncture with our biochemical findings in which a notable increase in these scavengers were observed in pathogen infected plants. Our aim was to find out differentially expressed genes in the resistant cultivar cDNA-SSH study. The EST data reported herein complement in detail to our previous studies of various *Brassica* sp studied. In conclusion, we have identified resistant candidate cDNAs from cabbage and 10 % of which were unclassified s being novel. We found a positive correlation in physio-biochemical and gene expression mechanisms in plant. The study has provided insights into the nature of pathogen-responsive genes in cabbage. The ESTs reported in the study can be used as candidate genes for developing molecular markers to assist breeding of black rot-resistant cabbage varieties. Defense-related genes have proven useful in cereals aimed at improving plants for increased disease resistance.

Research into *X. campestris* pv. *campestris* and closely related pathovars has now reached the genomic age, although it still lags behind the progress made from the investigation of *Pseudomonas* pathogens such as *P. syringae* pv. *tomato*. A number of disease resistance genes in Brassicas and *A. thaliana* have been postulated some have been mapped but none have been cloned. The effort to identify more disease resistance genes should be continued and some of the most important crops could be improved within the incorporation of disease resistance genes.

Efficient application of functional genomics tools for disease resistance can help us understand the plant defense signaling and could also reveal novel insights on the interactions between these signaling pathways (Chen et al. 2002). Furthermore, there is still an inadequacy of a reference set to be used as model for resistance genes that usually cluster in the genomic region. Our study has provided an insight into the host–pathogen interactions involved in resistant cabbage cultivar *Pusa mukta*–*X. campestris* pv. *campestris* first of its kind in the area of vegetable research. The study has also highlighted the prominent role of superoxide dismutase and catalase unigenes apart from peroxidase. The superoxide dismutase and catalase can be further utilized for marker-assisted breeding of cabbage cultivars resistant to the black rot pathogen.
